# Two new species of the bamboo-feeding genus *Buloria* from China (Hemiptera, Cicadellidae, Deltocephalinae)

**DOI:** 10.3897/zookeys.1277.186808

**Published:** 2026-04-16

**Authors:** Hai-Qing Sun, Qiang Luo, Zi-Zhong Li, Xiang-Sheng Chen, Lin Yang

**Affiliations:** 1 Guizhou Key Laboratory of Agricultural Biosecurity, Guizhou University, Guiyang, Guizhou, 550025, China Gannan Normal University Ganzhou China; 2 Institute of Entomology, Guizhou University, Guiyang, Guizhou, 550025, China Guizhou Key Laboratory of Agricultural Biosecurity, Guizhou University Guiyang China; 3 The Provincial Special Key Laboratory for Development and Utilization of Insect Resources of Guizhou, Guizhou University, Guiyang, Guizhou, 550025, China Institute of Entomology, Guizhou University Guiyang China; 4 Gannan Normal University, Ganzhou, Jiangxi, 341000, China The Provincial Special Key Laboratory for Development and Utilization of Insect Resources of Guizhou, Guizhou University Guiyang China

**Keywords:** Checklist, distribution, key, leafhopper, morphology, Oriental region, taxonomy

## Abstract

Two new species of the bamboo-feeding leafhopper genus *Buloria* Distant, 1908 (Cicadellidae, Deltocephalinae, Mukariini), *B. acuta* Sun, Li, Chen & Yang, **sp. nov**. and *B. falcata* Sun, Li, Chen & Yang, **sp. nov**. from Yunnan Province, China, are described and illustrated. A checklist of all known species of *Buloria* is provided, along with an identification key.

## Introduction

The leafhopper genus *Buloria* was established by Distant (1908) based on *Buloria gyponinoides* Distant, 1908 from India, and this species was earlier placed in the subfamily Nirvaninae (Oman et al. 1990). Viraktamath and Webb (2019) placed this genus in the tribe Mukariini of the subfamily Deltocephalinae (Hemiptera, Cicadellidae) and described two new species from India and Sri Lanka, respectively, *B. indica* Viraktamath & Webb, 2019 and *B. zeylanica* Viraktamath & Webb, 2019. Yang et al. (2022) reported this genus as a new record for the Chinese fauna, recording *B. gyponinoides* Distant, 1908 being found in Yunnan Province, southwestern China. It is the smallest genus within the tribe Mukariini, with only three described species, all of which are distributed in the Oriental region. This genus is similar to the genus *Mukaria*, from which it is mainly distinguished by the subgenital plates without macrosetae but with an apical membranous appendage, and male pygofer with a dorsal marginal short process near the attachment of segment X (Viraktamath and Webb 2019).

In this study, we describe and illustrate two new species of *Buloria* from Yunnan, southwestern China: *B. acuta* sp. nov. and *B. falcata* sp. nov., both collected on bamboo. Consequently, the genus *Buloria* now contains five species, three species recorded from China. A checklist and key based on morphological characteristics are provided to distinguish species.

## Materials and methods

The terminology of morphological and genital characters follows Viraktamath and Webb (2019). Body length was measured from the apex of the vertex to the tip of the forewing. All measurements are in millimeters (mm). External morphology was observed under a stereoscopic microscope, and characters were measured with an ocular micrometer. A KEYENCE VHX-1000E system was used to take photos of the adult habitus. Genitalia were prepared by immersing abdomens in a hot 10% NaOH solution for 3–5 min, then washing with water and immersing in glycerin jelly for observation and drawing using a Leica MZ 12.5 stereomicroscope. Illustrations were scanned with a CanoScan LiDE 200 and imported into Adobe Photoshop v. 6.0 for labeling and composition.

Type specimens examined are deposited in the Institute of Entomology, Guizhou University, Guiyang, Guizhou Province, China (**IEGU**).

## Taxonomy

### Genus Buloria

Taxon classificationAnimaliaHemipteraCicadellidae

Distant, 1908

B7A32A7C-799F-53CD-984D-2AC01A3220FC

Buloria Distant, 1908: 271; Viraktamath and Webb 2019: 10.

#### Type species.

*Buloria gyponinoides* Distant, 1908, by original designation.

#### Diagnosis.

External characters, as in generic description of Viraktamath and Webb (2019).

#### Distribution.

China, India, Sri Lanka.

##### Checklist and distributions of species of *Buloria* Distant, 1908

*B. acuta* Sun, Li, Chen & Yang, sp. nov.; China (Yunnan).

*B. falcata* Sun, Li, Chen & Yang, sp. nov.; China (Yunnan).

*B. gyponinoides* Distant, 1908; China (Yunnan), India (Andaman, Manipur, Tripura, West Bengal).

*B. indica* Viraktamath & Webb, 2019; India (Karnataka).

*B. zeylanica* Viraktamath & Webb, 2019; Sri Lanka (Galle).

##### Key to species of *Buloria* Distant, 1908

(modified from Viraktamath and Webb 2019)

**Table:** 

1	Forewing almost black; pygofer with process arising from posteroventral margin and somewhat dumbbell-shaped; aedeagal shaft with sparse tubercles (Viraktamath and Webb 2019: figs 1O, P, 19A, B, F)	***B. zeylanica* Viraktamath & Webb, 2019**
–	Forewing with white arcuate transverse stripes or bright yellow spots near the middle; pygofer without process arising from posteroventral margin and not dumbbell-shaped; ventral margin of aedeagal shaft without tubercles	**2**
2	Pygofer (Figs 6, 12) without process at subapical part of ventral margin or posterior margin; aedeagus (Fig. 10) with a short spinose process present on dorsal margin at curved position	***B. acuta* Sun, Li, Chen & Yang, sp. nov**.
–	Pygofer with process at subapical part of ventral margin or posterior margin; aedeagus without a short spinose process present on dorsal margin at curved position	**3**
3	Pygofer (Figs 18, 23) with acute angular process at middle part of ventral margin; aedeagal shaft (Fig. 20) long, sickle-shaped in lateral view	***B. falcata* Sun, Li, Chen & Yang, sp. nov**.
–	Pygofer without acute angular process at middle part of ventral margin; aedeagal shaft short, not sickle-shaped in lateral view	**4**
4	Aedeagal shaft with triangular lobe-like projection at midlength; pygofer dorsal process 0.50 as long as median length of pygofer (Viraktamath and Webb 2019: fig. 18A, C, D)	***B. indica* Viraktamath & Webb, 2019**
–	Aedeagal shaft without triangular lobe-like projection at midlength; pygofer dorsal process at most 0.25 as long as median length of pygofer (Viraktamath and Webb 2019: fig. 17C, D, F)	***B. gyponinoides* Distant, 1908**

### Buloria acuta

Taxon classificationAnimaliaHemipteraCicadellidae

Sun, Li, Chen & Yang sp. nov.

650704EF-8CF3-58AC-AC2F-CF38BD7F6B51

https://zoobank.org/7802D00D-D849-40FF-B224-57B3A7682559

Figs 1–12

#### Type materials.

***Holotype***: China • ♂: Yunnan Province, Yingjiang County, Nabang Town; 24°16'N, 97°57'E; sweeping, 18 August 2018; Qiang Luo and Hong-Xing Li leg.; IEGU. ***Paratypes***: China • 3♂♂, same collection data as for holotype; IEGU. 1♂, Yunnan Province, Yingjiang County, Nabang Town; 24°16'N, 97°57'E; sweeping, 30 May 2019; Xian-Yi Wang and Jia-Jia Wang leg.; IEGU.

#### Description.

***Measurements***. Total length: male 3.25–3.35 mm (*N* = 5).

**Figures 1–12.**
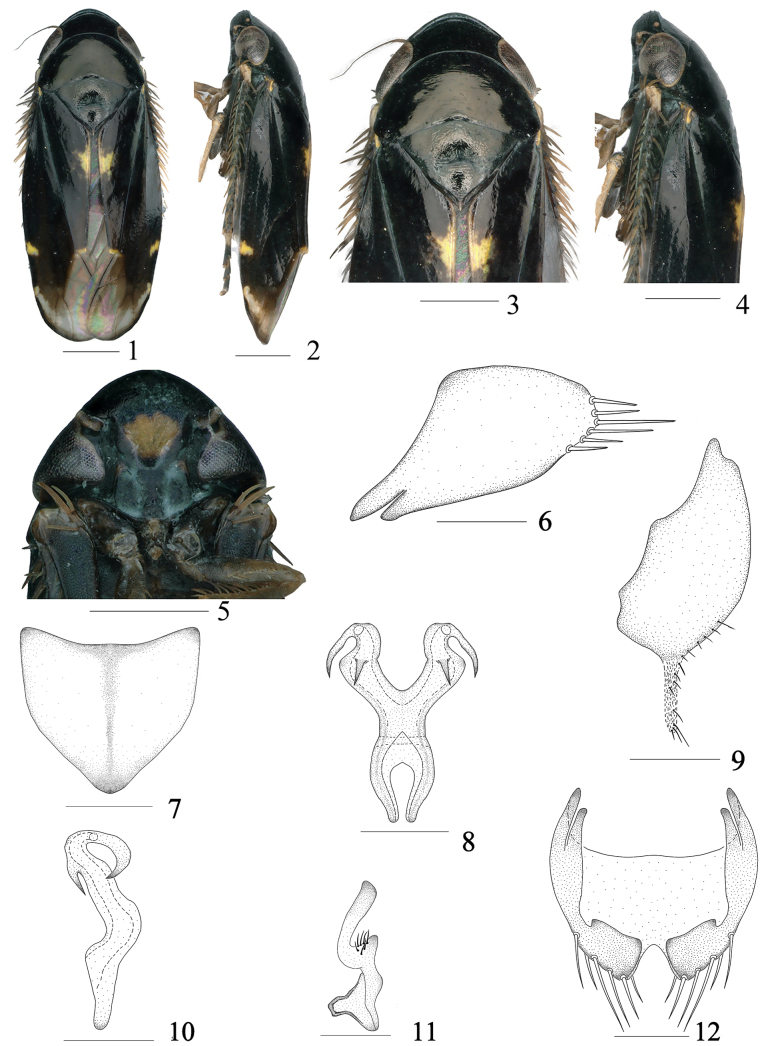
*Buloria acuta* Sun, Li, Chen & Yang, sp. nov. male. **1**. Habitus, dorsal view; **2**. Habitus, lateral view; **3**. Head and thorax, dorsal view; **4**. Head and thorax, lateral view; **5**. Face; **6**. Pygofer, lateral view; **7**. Valve, ventral view; **8**. Connective and aedeagus, dorsal view; **9**. Subgenital plate, lateral view; **10**. Connective and aedeagus, lateral view; **11**. Style; **12**. Pygofer, ventral view. Scale bars: 0.5 mm (**1–5**), 0.2 mm (**6–12**).

***Coloration***. Body (Figs 1, 2) mostly black. Crown (Figs 1–4) black. Eyes (Figs 1–5) dark brown. Ocelli (Figs 2, 4) brown. Face (Fig. 5) with irregular dark yellowish-brown marking medially, rest black. Pronotum and scutellum black (Fig. 3). Forewings (Figs 1, 2) mostly black, apical 1/3 light brown to hyaline, inner margin of clavus with bright yellow spot at middle part, basal part with small yellow spot, apical 1/3 of costal area with another bright yellow marking. Legs (Figs 1, 2) dark brown.

***Head and thorax***. Crown (Figs 1, 3) broad and short, midlength about 0.45 times inter ocular width, with marginal carinae and submarginal carinae. Ocelli (Figs 2, 4) located between the marginal carinae and submarginal carinae, close to apex, invisible from dorsal view. Face (Fig. 5) including eyes 1.5 times as wide as long. Base of frontoclypeus (Fig. 5) tumid, median to apical regions flat, with oblique wrinkles visible on surface. Anteclypeus (Fig. 5) 1.72 times as long as wide, median area distinctly elevated. Pronotum (Figs 1, 3) nearly equal in width to head including eyes, longer than crown in midline (1.80:1), finely punctate on the surface. Scutellum (Figs 1, 3) 1.51 times wider than long, longer than pronotum in midline (1.21:1). Transverse suture (Figs 1, 3) indistinct.

***Male genitalia***. Pygofer (Figs 6, 12) short, posterior margin with a row of thick and long bristles, ventral margin slightly curled inwardly, without distinct processes. Anterior margin of valve (Fig. 7) slightly concave, with width nearly equal to length. Subgenital plate (Fig. 9) narrow at base, gradually widening from median to apical parts, inner margin slightly undulate, without distinct setae, a membranous rod-like process at subapical part. Aedeagus (Figs 8, 10) thick at base, shafts diverging with laminate process, abruptly tapering at apical 1/3, curving ventrad, a short spinose process present on dorsal margin at curved position, then apex directed dorsad and extends towards the middle. Connective (Figs 8, 10) U-shaped, completely fused with aedeagus. Style (Fig. 11) broad at base, constricted medially, apex fingerlike, gradually widened at apex, obliquely directed outward, with several fine setae subapically.

#### Distribution.

China (Yunnan).

#### Host plant.

*Dendrocalamus hamiltonii* Nees & Arn. ex Munro (Poales: Poaceae: Bambusoideae).

#### Etymology.

The species name is derived from the Latin word “*acuta*”, referring to the aedeagus with two small sharp spinous processes in the middle part.

#### Remarks.

This species is similar to *B. falcata* Sun, Li, Chen & Yang, sp. nov., but differs from the latter in: (1) ventral and posterior margins of pygofer without a spinous process (ventral margin of pygofer with an acute angle, posterior margin with a slender fingerlike process in *B. falcata*); (2) a short spinose process present on the dorsal margin at the curved position of the aedeagus (an irregular lamellar process present on dorsal margin at curved position of aedeagus in *B. falcata*); and (3) aedeagal shaft curved and extends towards the middle of aedeagus (aedeagal shaft curved and extends towards the base of aedeagus in *B. falcata*).

### Buloria falcata

Taxon classificationAnimaliaHemipteraCicadellidae

Sun, Li, Chen & Yang sp. nov.

232DCBBC-F135-5460-BFFE-B96329316590

https://zoobank.org/A84E03D9-A79B-4EB4-90E7-8617BDBA7DD6

Figs 13–24

#### Type material.

***Holotype***: China • ♂; Yunnan Province, Mengla County; 21°47'N, 101°57'E; sweeping, 27 August 2017; Qiang Luo and Nian Gong leg.; IEGU. ***Paratypes***: China • 2♂♂; same collection data as for holotype; IEGU.

#### Description.

***Measurements***. Total length: male 3.45–3.58 mm (*N* = 3).

**Figures 13–24.**
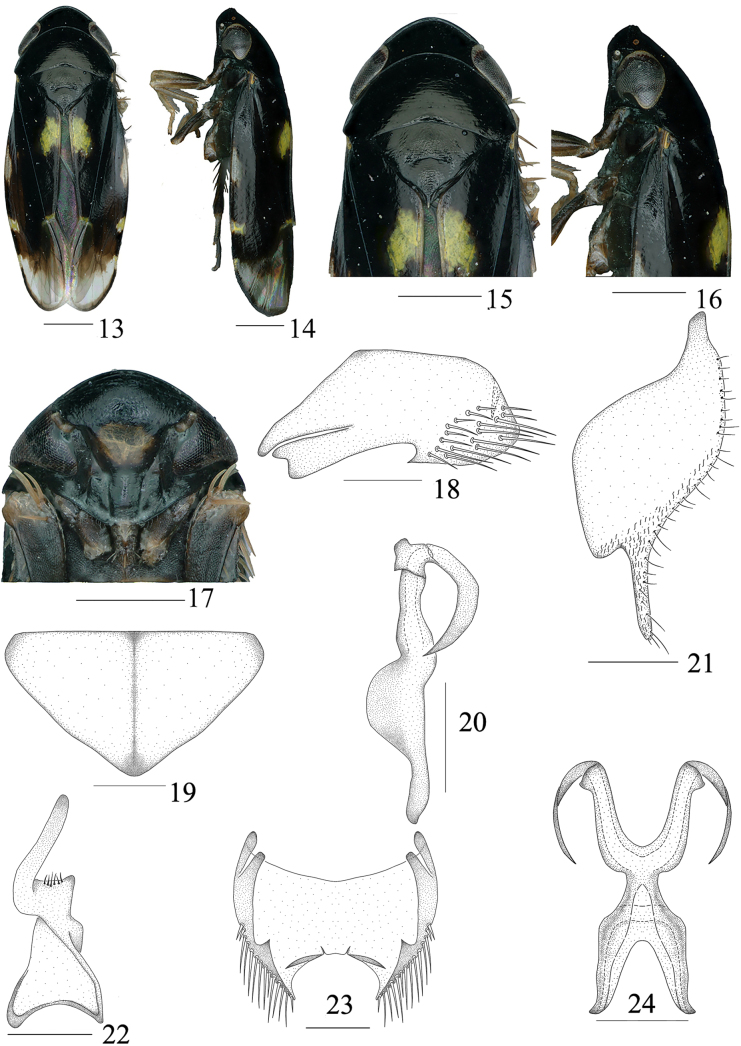
*Buloria falcata* Sun, Li, Chen & Yang, sp. nov. male. **13**. Habitus, dorsal view; **14**. Habitus, lateral view; **15**. Head and thorax, dorsal view; **16**. Head and thorax, lateral view; **17**. Face; **18**. Pygofer, lateral view; **19**. Valve, ventral view; **20**. Connective and aedeagus, lateral view; **21**. Subgenital plate, lateral view; **22**. Style; **23**. Pygofer, ventral view; **24**. Connective and aedeagus, dorsal view. Scale bars: 0.5 mm (**13–17**), 0.2 mm (**18–24**).

***Coloration***. Body (Figs 13, 14) mostly black and shiny. Crown (Figs 13–16) black. Eyes (Figs 13–17) dark brown. Face (Fig. 17) with roughly rectangular yellowish-brown marking medially. Forewings (Figs 13, 14) mostly black, apical 1/3 light brown, clavus with irregular bright yellow marking medially, apex with small irregular dark yellow spot, costal area with two irregular markings, one white, slightly transparent spot at basal 1/3, another irregular bright yellow spot on subapical costal area. Legs (Figs 13, 14) dark brown to yellowish-brown.

***Head and thorax***. Crown (Figs 13, 15) with anterior margin rounded and convex, midlength about 0.44 times inter ocular width, with 2–3 marginal carinae and submarginal carinae. Ocelli (Figs 14, 16) located between the marginal carinae and submarginal carinae, close to apex, invisible from dorsal view. Face (Fig. 17) including eyes 1.47 times as wide as long, with weak folds; base of frontoclypeus tumid and protuberant, gradually flattened from median to apical parts, with oblique wrinkles visible on surface. Middle length of anteclypeus slightly wider than its two ends, median area distinctly elevated. Pronotum (Figs 13, 15) slightly wider than head including eyes, longer than median length of crown (1.95: 1), finely punctate on the surface. Scutellum (Figs 13, 15) 1.42 times wider than long, longer than pronotum in midline (1.24: 1), basal margin longer than lateral margins. Transverse suture (Figs 13, 15) indistinct.

***Male genitalia***. Pygofer (Figs 18, 23) broad at base, ventral margin with acute angular process at middle part, posterior margin with fingerlike sharp process at subapical part, directed to both sides, apical area with thick and long bristles. Valve (Fig. 19) obtusely triangular. Subgenital plate (Fig. 21) broad, with numerous slender small setae at lateral area, a membranous rod-like process at subapical part. Aedeagus (Figs 20, 24) robust, falcate in lateral view, shafts diverging with laminate process, each shaft with an irregular sclerite at middle part, apical 1/2 curved, then apex directed dorsad, extends towards base. Connective (Figs 20, 24) U-shaped, fused with aedeagus. Style (Fig. 22) broad at base, narrower at middle part, apical part elongated into a long fingerlike process, obliquely directed outward.

#### Distribution.

China (Yunnan).

#### Host plant.

Bambusoideae sp. (Poales: Poaceae: Bambusoideae).

#### Etymology.

The species name is derived from the Latin word “*falcata*”, referring to the aedeagus falcate in lateral view.

#### Remarks.

This new species is similar to *B. acuta* Sun, Li, Chen & Yang, sp. nov., but differs from the latter in: (1) ventral margin of pygofer with an acute angular process (ventral margin of pygofer without acute angular process in *B. acuta*); (2) shafts of aedeagus with an irregular sclerite at middle part (shafts of aedeagus without irregular sclerite at middle part in *B. acuta*); and (3) aedeagal shaft curved and extends towards the base of the aedeagus (aedeagal shaft curved and extends towards the middle of aedeagus in *B. acuta*).

## Discussion

Geographically, all known species of the genus *Buloria* Distant, 1908 are restricted to the Oriental region (Viraktamath and Webb 2019; Yang et al. 2022). Prior to this study, only three species had been described in this genus. Herein, we expand the genus to five described species, three of which are recorded in China, and all Chinese *Buloria* species are distributed in Yunnan Province. Owing to its complex terrain, an extreme altitudinal gradient ranging from 76 to over 6700 m, and the convergence of the East Asian, South Asian and Qinghai-Tibet Plateau monsoons, it has diverse climatic types with distinct regional variations in temperature and precipitation. The unique topographic position and climatic diversity of Yunnan have fostered exceptional biodiversity, and we anticipate that additional field surveys in Yunnan and elsewhere in China will reveal further undescribed species within this genus.

All host records of Mukariini available to date indicate that all species feed and reproduce exclusively on bamboos (Viraktamath and Viraktamath 1996; Khatri and Webb 2011; Chen et al. 2012; Luo et al. 2018a, b, 2019; Viraktamath and Webb 2019; Zhao et al. 2023). The two *Buloria* species treated herein likewise utilize bamboo as host plants: *B. acuta* sp. nov. was collected on *Dendrocalamus hamiltonii* Nees & Arn. ex Munro, and *B. falcata* sp. nov. was taken from an unidentified Bambusoideae species. Because bamboos are evergreen and constitute a valuable renewable resource—with applications in environmental greening, packaging, food processing, and eco-friendly furniture and construction—precise identification of the leafhopper taxa capable of inflicting damage on them is an essential prerequisite for effective quarantine measures and sustainable management strategies (Chen and Yang 2024; Zhao et al. 2024).

## Supplementary Material

XML Treatment for Buloria

XML Treatment for Buloria acuta

XML Treatment for Buloria falcata

## Additional information

### Artificial Intelligence (AI) use

The authors accept full responsibility for the content of the manuscript, including the disclosure of any use of AI.

No AI tools were used in the preparation of this manuscript.

### Author ORCIDs

H.-Q. Sun https://orcid.org/0009-0004-4576-4132

Q. Luo https://orcid.org/0000-0001-9927-1887

Z.-Z. Li https://orcid.org/0000-0003-1609-9992

X.-S. Chen https://orcid.org/0000-0001-9801-0343

L. Yang https://orcid.org/0000-0002-7841-5156

### Data availability

All of the data that support the findings of this study are available in the main text.
